# Supplementary material for: Measurement of China’s green development level and its spatial differentiation in the context of carbon neutrality
Source: PLoS One. 2023 Apr 10;18(4):e0284207. doi: 10.1371/journal.pone.0284207 (PMC10085045; doi:10.1371/journal.pone.0284207)
Supplement: S1 File — (DOCX) [file pone.0284207.s001.docx]

Corresponding data of Fig. 2

| year | Green Development Level | Green Wealth Index | Green Growth Index | Green Welfare Index |
| --- | --- | --- | --- | --- |
| 2010 | 0.2469 | 0.2621 | 0.1594 | 0.4567 |
| 2011 | 0.2548 | 0.2546 | 0.1728 | 0.4780 |
| 2012 | 0.2735 | 0.2631 | 0.1916 | 0.4876 |
| 2013 | 0.2968 | 0.2819 | 0.2064 | 0.4984 |
| 2014 | 0.3065 | 0.2809 | 0.2233 | 0.5002 |
| 2015 | 0.3205 | 0.2803 | 0.2432 | 0.5122 |
| 2016 | 0.3411 | 0.2830 | 0.2802 | 0.4990 |
| 2017 | 0.3557 | 0.2734 | 0.3073 | 0.5201 |
| 2018 | 0.3724 | 0.2816 | 0.3311 | 0.5087 |
| 2019 | 0.3848 | 0.2829 | 0.3454 | 0.5223 |

Corresponding data of Fig. 3

| Green Development Level | | |  |  |
| --- | --- | --- | --- | --- |
| year | Eastern Region | Central Region | Western Region | Northeast Region |
| 2010 | 0.2735 | 0.1826 | 0.2302 | 0.3480 |
| 2011 | 0.2882 | 0.1905 | 0.2386 | 0.3312 |
| 2012 | 0.3063 | 0.2028 | 0.2586 | 0.3603 |
| 2013 | 0.3434 | 0.2165 | 0.2682 | 0.4068 |
| 2014 | 0.3470 | 0.2308 | 0.2848 | 0.4025 |
| 2015 | 0.3713 | 0.2450 | 0.2916 | 0.4087 |
| 2016 | 0.4023 | 0.2663 | 0.3060 | 0.4157 |
| 2017 | 0.4205 | 0.2788 | 0.3228 | 0.4142 |
| 2018 | 0.4482 | 0.2855 | 0.3360 | 0.4275 |
| 2019 | 0.4592 | 0.3029 | 0.3492 | 0.4304 |

| Green Wealth Index | |  |  |  |
| --- | --- | --- | --- | --- |
| year | Eastern Region | Central Region | Western Region | Northeast Region |
| 2010 | 0.2312 | 0.2178 | 0.2705 | 0.4225 |
| 2011 | 0.2246 | 0.2045 | 0.2648 | 0.4172 |
| 2012 | 0.2288 | 0.2169 | 0.2740 | 0.4299 |
| 2013 | 0.2613 | 0.2141 | 0.2873 | 0.4666 |
| 2014 | 0.2581 | 0.2180 | 0.2884 | 0.4549 |
| 2015 | 0.2571 | 0.2226 | 0.2844 | 0.4580 |
| 2016 | 0.2609 | 0.2290 | 0.2844 | 0.4596 |
| 2017 | 0.2434 | 0.2145 | 0.2831 | 0.4551 |
| 2018 | 0.2499 | 0.2195 | 0.2957 | 0.4603 |
| 2019 | 0.2475 | 0.2221 | 0.2935 | 0.4831 |

| Green Growth Index | |  |  |  |
| --- | --- | --- | --- | --- |
| year | Eastern Region | Central Region | Western Region | Northeast Region |
| 2010 | 0.2332 | 0.1046 | 0.1371 | 0.1046 |
| 2011 | 0.2564 | 0.1099 | 0.1474 | 0.1133 |
| 2012 | 0.2761 | 0.1320 | 0.1631 | 0.1331 |
| 2013 | 0.2961 | 0.1418 | 0.1744 | 0.1540 |
| 2014 | 0.3153 | 0.1602 | 0.1932 | 0.1535 |
| 2015 | 0.3469 | 0.1783 | 0.2098 | 0.1495 |
| 2016 | 0.4048 | 0.2095 | 0.2337 | 0.1768 |
| 2017 | 0.4448 | 0.2334 | 0.2553 | 0.1880 |
| 2018 | 0.4878 | 0.2537 | 0.2651 | 0.2054 |
| 2019 | 0.5152 | 0.2641 | 0.2751 | 0.1996 |

| Green Welfare Index | |  |  |  |
| --- | --- | --- | --- | --- |
| year | Eastern Region | Central Region | Western Region | Northeast Region |
| 2010 | 0.4698 | 0.4414 | 0.4169 | 0.5896 |
| 2011 | 0.4935 | 0.5007 | 0.4437 | 0.5075 |
| 2012 | 0.5102 | 0.4617 | 0.4647 | 0.5484 |
| 2013 | 0.5296 | 0.5054 | 0.4399 | 0.5951 |
| 2014 | 0.5042 | 0.5092 | 0.4614 | 0.6113 |
| 2015 | 0.5321 | 0.5116 | 0.4595 | 0.6397 |
| 2016 | 0.5075 | 0.5065 | 0.4608 | 0.5952 |
| 2017 | 0.5337 | 0.5406 | 0.4825 | 0.5718 |
| 2018 | 0.5258 | 0.5030 | 0.4793 | 0.5707 |
| 2019 | 0.5096 | 0.5455 | 0.5183 | 0.5336 |

Corresponding data of Fig.4

| province | Green Development Level | Green Wealth Index | Green Growth Index | Green Welfare Index |
| --- | --- | --- | --- | --- |
| Beijing | 0.5363 | 0.0831 | 0.7648 | 0.6685 |
| Tianjin | 0.3555 | 0.1695 | 0.4196 | 0.5223 |
| Hebei | 0.1857 | 0.0993 | 0.2062 | 0.5282 |
| Shanxi | 0.1876 | 0.0995 | 0.1966 | 0.5610 |
| Inner Mongolia | 0.4914 | 0.5241 | 0.2240 | 0.6233 |
| Liaoning | 0.3235 | 0.3002 | 0.2416 | 0.4723 |
| Jilin | 0.3959 | 0.4360 | 0.1946 | 0.5283 |
| Heilongjiang | 0.5720 | 0.7131 | 0.1627 | 0.6002 |
| Shanghai | 0.8499 | 0.5773 | 0.8099 | 0.5288 |
| Jiangsu | 0.5731 | 0.2991 | 0.6330 | 0.5589 |
| Zhejiang | 0.4915 | 0.2681 | 0.5678 | 0.4565 |
| Anhui | 0.3036 | 0.2162 | 0.2622 | 0.5707 |
| Fujian | 0.4005 | 0.2984 | 0.3859 | 0.4418 |
| Jiangxi | 0.3576 | 0.3692 | 0.2302 | 0.4563 |
| Shandong | 0.2909 | 0.1281 | 0.3227 | 0.6076 |
| Henan | 0.2310 | 0.0989 | 0.2219 | 0.6890 |
| Hubei | 0.3767 | 0.2449 | 0.3643 | 0.5454 |
| Hunan | 0.3609 | 0.3041 | 0.3093 | 0.4503 |
| Guangdong | 0.5263 | 0.2498 | 0.6753 | 0.3818 |
| Guangxi | 0.3000 | 0.3156 | 0.2299 | 0.3549 |
| Hainan | 0.3828 | 0.3026 | 0.3666 | 0.4016 |
| Chongqing | 0.3247 | 0.2169 | 0.3236 | 0.5014 |
| Sichuan | 0.4362 | 0.3067 | 0.3932 | 0.5570 |
| Guizhou | 0.2685 | 0.2328 | 0.2455 | 0.4114 |
| Yunnan | 0.4194 | 0.3390 | 0.3396 | 0.5304 |
| Shaanxi | 0.3134 | 0.1873 | 0.2648 | 0.6895 |
| Gansu | 0.3016 | 0.2256 | 0.2238 | 0.6349 |
| Qinghai | 0.5731 | 0.5699 | 0.3829 | 0.4387 |
| Ningxia | 0.1647 | 0.0957 | 0.1936 | 0.4786 |
| Xinjiang | 0.2488 | 0.2151 | 0.2048 | 0.4806 |
| mean | 0.3848 | 0.2829 | 0.3454 | 0.5223 |

**Corresponding data of Fig.5**

Regional differences of China's green development level from 2010 to 2019

| Green Development Level | Eastern Region | Central Region | Western Region | Northeast Region |
| --- | --- | --- | --- | --- |
| 2010 | 0.0706 | 0.0847 | 0.0831 | 0.0318 |
| 2011 | 0.0703 | 0.0845 | 0.0739 | 0.0274 |
| 2012 | 0.0516 | 0.0570 | 0.0716 | 0.0150 |
| 2013 | 0.0562 | 0.0389 | 0.0779 | 0.0173 |
| 2014 | 0.0609 | 0.0415 | 0.0640 | 0.0230 |
| 2015 | 0.0572 | 0.0496 | 0.0564 | 0.0262 |
| 2016 | 0.0558 | 0.0507 | 0.0590 | 0.0248 |
| 2017 | 0.0631 | 0.0441 | 0.0533 | 0.0199 |
| 2018 | 0.0602 | 0.0365 | 0.0583 | 0.0241 |
| 2019 | 0.0701 | 0.0291 | 0.0518 | 0.0287 |

Regional differences of China's green wealth index from 2010 to 2019

| Green Wealth Index | Eastern Region | Central Region | Western Region | Northeast Region |
| --- | --- | --- | --- | --- |
| 2010 | 0.1340 | 0.1001 | 0.0833 | 0.0498 |
| 2011 | 0.1302 | 0.0873 | 0.0863 | 0.0543 |
| 2012 | 0.1238 | 0.1030 | 0.0933 | 0.0537 |
| 2013 | 0.1305 | 0.0834 | 0.0916 | 0.0465 |
| 2014 | 0.1335 | 0.0850 | 0.0917 | 0.0535 |
| 2015 | 0.1288 | 0.0878 | 0.0837 | 0.0517 |
| 2016 | 0.1334 | 0.0874 | 0.0840 | 0.0599 |
| 2017 | 0.1543 | 0.0967 | 0.0942 | 0.0539 |
| 2018 | 0.1399 | 0.0867 | 0.1021 | 0.0598 |
| 2019 | 0.1437 | 0.1059 | 0.1022 | 0.0622 |

Regional differences of China's green growth index from 2010 to 2019

| Green Growth Index | Eastern Region | Central Region | Western Region | Northeast Region |
| --- | --- | --- | --- | --- |
| 2010 | 0.0802 | 0.0924 | 0.0884 | 0.0201 |
| 2011 | 0.0866 | 0.0707 | 0.0503 | 0.0351 |
| 2012 | 0.0709 | 0.0630 | 0.0513 | 0.0384 |
| 2013 | 0.0648 | 0.0537 | 0.0440 | 0.0315 |
| 2014 | 0.0653 | 0.0559 | 0.0386 | 0.0237 |
| 2015 | 0.0707 | 0.0517 | 0.0394 | 0.0159 |
| 2016 | 0.0611 | 0.0468 | 0.0370 | 0.0149 |
| 2017 | 0.0682 | 0.0343 | 0.0301 | 0.0123 |
| 2018 | 0.0684 | 0.0263 | 0.0291 | 0.0121 |
| 2019 | 0.0728 | 0.0227 | 0.0303 | 0.0131 |

Regional differences of China's green welfare index from 2010 to 2019

| Green Welfare Index | Eastern Region | Central Region | Western Region | Northeast Region |
| --- | --- | --- | --- | --- |
| 2010 | 0.0408 | 0.0167 | 0.0265 | 0.0076 |
| 2011 | 0.0221 | 0.0047 | 0.0232 | 0.0058 |
| 2012 | 0.0137 | 0.0265 | 0.0301 | 0.0023 |
| 2013 | 0.0222 | 0.0182 | 0.0284 | 0.0038 |
| 2014 | 0.0195 | 0.0091 | 0.0202 | 0.0019 |
| 2015 | 0.0214 | 0.0133 | 0.0134 | 0.0046 |
| 2016 | 0.0240 | 0.0111 | 0.0329 | 0.0020 |
| 2017 | 0.0376 | 0.0166 | 0.0245 | 0.0015 |
| 2018 | 0.0090 | 0.0061 | 0.0248 | 0.0029 |
| 2019 | 0.0142 | 0.0106 | 0.0175 | 0.0048 |

**Corresponding data of Fig.6**

Local spatial clustering table of China's green wealth index in 2010 and 2019

|  | HH | LH | LL | HL |
| --- | --- | --- | --- | --- |
| 2010 | Liaoning, Jilin, Heilongjiang, Zhejiang, Fujian, Guangxi, | Guangong, Guizhou, Gansu, | Beijing, Tianjin, Hebei, Shanxi, Jiangsu, Anhui, Shandong, Henan, Hubei, Chongqing, Shaanxi, Ningxia, Xinjiang | Inner Mongolia, Shanghai, Jiangxi, Hunan, Hainan, Sichuan, Yunnan, Qinhai, |
| 2019 | Liaoning, Jilin, Heilongjiang, Fujian, | Zhejiang, Guangong, Gansu, | Beijing, Tianjin, Hebei, Shanxi, Anhui, Shandong, Henan, Hubei, Chongqing, Guizhou, Shaanxi, Ningxia, Xinjiang | Inner Mongolia, Shanghai, Jiangsu, Jiangxi, Hunan, Guangxi, Hainan, Sichuan, Yunnan, Qinhai, |

Local spatial clustering table of China's green growth index in 2010 and 2019

|  | HH | LH | LL | HL |
| --- | --- | --- | --- | --- |
| 2010 | Tianjin, Shanghai, Jiangsu, Zhejiang, Fujian, Guangong, Hainan, | Anhui, Jiangxi, Shandong, Hunan, Guangxi, Guizhou, Gansu, | Hebei, Shanxi, Inner Mongolia, Liaoning, Jilin, Heilongjiang, Henan, Shaanxi, Ningxia, Xinjiang | Beijing, Hubei, Chongqing, Sichuan, Yunnan, Qinhai, |
| 2019 | Tianjin, Shanghai, Jiangsu, Zhejiang, Fujian, Hubei, Hainan, | Hebei, Anhui, Jiangxi, Shandong, Henan, Hunan, Guangxi, Guizhou, | Shanxi, Inner Mongolia, Liaoning, Jilin, Heilongjiang, Chongqing, Yunnan, Shaanxi, Gansu, Ningxia, Xinjiang | Beijing, Guangong, Sichuan, Qinhai, |

Local spatial clustering table of China's green welfare index in 2010 and 2019

|  | HH | LH | LL | HL |
| --- | --- | --- | --- | --- |
| 2010 | Beijing, Tianjin, Hebei, Shanxi, Inner Mongolia, Liaoning, Jilin, Heilongjiang, Shanghai, Shandong, Henan, | Zhejiang, Hubei, Ningxia, | Anhui, Fujian, Hunan, Guangong, Guangxi, Hainan, Sichuan, Guizhou, Yunnan, Gansu, Qinhai, | Jiangsu, Jiangxi, Chongqing, Shaanxi, Xinjiang |
| 2019 | Beijing, Hebei, Shanxi, Inner Mongolia, Jilin, Heilongjiang, Jiangsu, Anhui, Shandong, Henan, Shaanxi, | Tianjin, Liaoning, Zhejiang, Qinhai, Ningxia, Xinjiang | Fujian, Jiangxi, Hunan, Guangong, Guangxi, Hainan, Chongqing, Guizhou, | Shanghai, Hubei, Sichuan, Yunnan, Gansu, |
