# Supplementary material for: Measurement of China’s green development level and its spatial differentiation in the context of carbon neutrality
Source: PLoS One. 2023 Apr 10;18(4):e0284207. doi: 10.1371/journal.pone.0284207 (PMC10085045; doi:10.1371/journal.pone.0284207)
Supplement: Attachment — Submitted filename: Response to Reviewers.docx [file pone.0284207.s003.docx]

Dear Reviewers.

I am very grateful to the two reviewers for their constructive comments, and I am doubly honored. In response to your comments, I have revised this paper accordingly.

First, about the data used in the submission of this paper. I have organized all the data in the indicator system of this paper in a master table, which also contains the calculation results of the green development level and its component indices as well as pictures. The raw data involved in this paper are organized in separate tables and categorized in different files. Moreover, the links to the data sources have been updated in this paper so that readers can use them directly. In order to increase the openness of the calculation methods of the indicators, the calculation methods and corresponding indicators of Green Total Factor Productivity (GTFP) are included in the notes at the bottom of Table 1. Considering China's accession to WTO in 2003, the GDP data used to calculate GTFP are deflated with 2004 as the base period, and the amount of fixed asset investment is deflated with 2004 as the base period for the price index. It should be noted here that the same method of deflating other years is also desirable.

Second, regarding the need for the introduction to describe the efforts of public authorities in other countries in the transition to a low-carbon economy and green development. This paper briefly mentions the carbon reduction practices in the United States, Japan, and Korea in the introduction, and introduces the carbon reduction instruments and experiences proposed by the European Union and its published *European Green Deal* to enhance the necessity and importance of choosing carbon neutrality as the context for this study.

Third, about adding the discussion section. I have added a new section, Discussion, specifically to this paper. This section first selected two similar studies published in recent years with Chinese provinces as samples, and compared the indicator system and conclusions from these two articles with the results of my study. In terms of the main conclusions, my study is identical or similar to the conclusions of these two studies. In terms of some of the more detailed conclusions, there are major or minor differences between these studies, which are due to the differences in the indicators these studies included in the green development level indicator system and the different methods used to measure the green development level. At the end of the discussion section, I still pointed out the limitations of this study and the direction of future research.

Fourth, about increasing the references cited in various parts of the paper. In order to enhance the persuasive power of the ideas and academic standardization of this paper, the introduction and discussion of this paper further increase the citations in related studies, especially in the discussion section of this paper also cites a lot of other related studies as the supporting evidence of this paper's view. In addition, the original manuscript involved a lot of Chinese literature, which has been partially replaced with similar literature from international journals and has been specifically marked out in this revision.

Thank you and best regards.

Yours sincerely,

Dr. Kun LIANG

March 12, 2023
